# The Prescription of Drug Ontology 2.0 (PDRO): More Than the Sum of Its Parts

**DOI:** 10.3390/ijerph182212025

**Published:** 2021-11-16

**Authors:** Jean-François Ethier, François Goyer, Paul Fabry, Adrien Barton

**Affiliations:** 1 Groupe de Recherche Interdisciplinaire en Informatique de la Santé (GRIIS.ca), Université de Sherbrooke, Sherbrooke, QC J1K 2R1, Canada; francois.goyer@usherbrooke.ca (F.G.); paul.fabry@usherbrooke.ca (P.F.); Adrien.Barton@irit.fr (A.B.); 2 IRIT—Institut de Recherche en Informatique de Toulouse (CNRS), 31400 Toulouse, France

**Keywords:** drug ontology, drug prescription, optimization of prescription, medication management, ontology development, data annotation

## Abstract

While drugs and related products have profoundly changed the lives of people around the world, ongoing challenges remain, including inappropriate use of a drug product. Inappropriate uses can be explained in part by ambiguous or incomplete information, for example, missing reasons for treatments, ambiguous information on how to take a medication, or lack of information on medication-related events outside the health care system. In order to fully assess the situation, data from multiple systems (electronic medical records, pharmacy and radiology information systems, laboratory management systems, etc.) from multiple organizations (outpatient clinics, hospitals, long-term care facilities, laboratories, pharmacies, registries, governments) on a large geographical scale is needed. Formal knowledge models like ontologies can help address such an information integration challenge. Existing approaches like the Observational Medical Outcomes Partnership are discussed and contrasted with the use of ontologies and systems using them for data integration. The PRescription Drug Ontology 2.0 (PDRO 2.0) is then presented and entities that are paramount in addressing this problematic are described. Finally, the benefits of using PDRO are discussed through a series of exemplar situation.

## 1. Introduction

Drugs and related products have profoundly changed the lives of people around the world, and new options are being discovered regularly. This is true for disease prevention (vaccines, vitamins), acute illnesses (antibiotics, antithrombotics) or chronic ailments (insulin, hypotensive agents).

Notwithstanding new discoveries, this new drug era is accompanied by two ongoing challenges: inappropriate use of drugs or missed opportunities to use a drug product to decrease morbidity or mortality. Obviously, the ways health systems are organized will vary across jurisdictions and will influence these situations. Let us consider some concrete examples of these challenges.

Inappropriate usage can stem from multiple causes. The first to be explored is the **incomplete or ambiguous information** on a prescription:

### 1.1. Ibuprofen 600 mg PO TID x 2 Weeks

High dose non-steroidal anti-inflammatory drugs (NSAIDs), like those containing ibuprofen, can be used for multiple reasons, including musculoskeletal conditions like back or hip osteoarthritis, but it can also be used to treat other inflammatory conditions like pericarditis. NSAIDs are also relatively contraindicated in kidney diseases [1] (acute or chronic). A care provider would very likely elect not to use them for musculoskeletal reasons in the presence of kidney disease but might very well decide to use them nonetheless for pericarditis [2]. The problem is that the indication (the reason) for the prescription is often not present on it. This hinders the pharmacist’s ability to fully play his role and stop potentially inappropriate prescriptions leading to harm for patients.

*A prescription containing this can be associated with a patient receiving too high a dose leading to renal problems, bleeding or cardiac conditions without a favorable risk-benefit ratio.*

### 1.2. Gluconorm 2 mg PO TID

When prescribing a treatment, physicians often express the need for a particular active ingredient. This is because many drug product preparations have that same active ingredient and are often largely equivalent, not to mention it is difficult if not impossible to know what exact brand will be available in the pharmacy where the patient will obtain the medication for a prescription.

The problem arises when care providers sometimes use a commercial brand name on a prescription but with the intent to refer to any drug with the same active ingredient. This is because this name is often short, simpler and ingrained in memory as it was the first one on the market. 

On the other hand, including a commercial name might in some cases indicate a true desire to prescribe this specific drug to avoid an allergy [3] or simply because a previous trial has shown that this patient does not react well to some drug products but can tolerate this specific product.

How can the pharmacist know what to serve the patient, especially since many jurisdictions have regulations [4] in place to automatically substitute a cheaper alternative to a brand-name drug product?

*This can lead to a patient receiving medication that is less effective for him/her, to which s/he is allergic, or which entails unnecessary costs (to the patient, the insurer or both).*

### 1.3. Cardizem 60 mg PO QID

It is often the case that the information transmitted by healthcare providers is not sufficient to safely guide the patient’s drug-taking processes. In the case above, the physician has written “QID” (standing for the Latin expression “quater in die”. This is unlikely to be understood by the patient, but it will often have been said to take the medication four times a day orally by the physician. Alas, a large proportion of recommendations (less than 50% without prompting) are not remembered by the patients following a visit [5].

The pharmacist will provide a label on a medication container or some other documentation, for example, from the manufacturer. This label may say something such as “Cardizem 30 mg tab, take 2 tabs 4 times a day”. Yet, this leaves a fair bit of interpretation to the patient, who is the actor with the least knowledge in the field (compared to the physician or the pharmacist). A patient, unaware of the pharmacokinetics of Cardizem, might interpret these instructions as four times between 9 a.m. and 6 p.m. (the time of his other evening medication) instead of every six hours (as intended by his physician). His physician inquiring about the patient’s average morning pulse might be surprised that it is still high. As a result, the physician increases his dose even though it is acceptable on average.

*The patient subsequently develops bradycardia as the medication is increased too much.*

In a context where a patient has received medication but the complete physician’s instructions, as written on the prescription, cannot be accessed, **false assumptions based on other information sources, especially distribution records from pharmacists** can lead to adverse events.

### 1.4. Amlodipine 5 mg PO Once a Day Increased by the Family Physician to Amlodipine 10 mg PO Once a Day

In this case, right after getting his monthly pills from the pharmacy, a patient on 5 mg daily saw his family physician who, given his persistent high blood pressure decided to increase (double) his dose to 10 mg daily. 

The patient took the prescription to the pharmacy. At that point, he had a standing order of distributions for 30 tabs containing 5 mg of amlodipine every 30 days with six renewals left. When the patient gave the new prescription, the existing distribution order was terminated and labeled as inactive. In order to save some money for the patient, the pharmacist told him to simply take 2 pills of 5 mg for two weeks and then come back to the pharmacy to start the 10 mg pills treatment. That led to a distribution order of 10 mg pills but starting only in 15 days.

When the patient visits the emergency room at night in a state of confusion and weakness because of low blood pressure, the emergency physician might look at the pharmacy information because the patient is confused. It will then seem that the patient is not taking any amlodipine at this point in time, since the old distribution order has been discontinued and the new one has not been activated yet.

*This could lead to incorrect and delayed diagnoses and other adverse events.*

### 1.5. Clopidogrel 75 mg PO Once a Day, 30 Days x 12

Clopidogrel is routinely given for a duration of 12 months following a percutaneous transluminal coronary angioplasty (PTCA) to avoid stent thrombosis [6]. As it is given in conjunction with Aspirin (another antithrombotic), the bleeding risks are substantial, and a double therapy should be given only for the minimal time required to avoid a stent thrombosis. In other cases, like strokes, clopidogrel would be a long-term treatment without a specified end date.

A prescription to prevent further strokes might need to be renewed for a relatively long time in this case, but not if it is for a PTCA. At least two sets of circumstances might lead to prolonged clopidogrel administration beyond what is required, potentially leading to avoidable bleeding. Firstly, the initial prescription did not include an indication. In rarer occasions, the clinical indication (reason) for the treatment might have been included in the prescription (“post PTCA”) or it might have even included a note stating to “stop after 12 months”. 

Secondly, even if it did include an indication, the pharmacy systems (or in some countries the insurer’s system) will often only record information about their processes. This information includes the instructions given to the patient by the pharmacist, for example “Take Plavix 75 mg tab once daily in the morning”, as well as the dispensing information (e.g., “Distributed 9 January 2020, 11/12 distributions remaining”).

It is not rare for other care providers to take over the care of patients in the community after a hospital stay, especially when specialized care is provided far from home, sometimes in other jurisdictions. In this context, a family care physician might be asked by a pharmacist what to do with the clopidogrel which was prescribed a few months back by another physician. Without the original prescription (assuming it contained the indication or at least precise treatment duration intent), wrong assumptions can occur and clopidogrel can be extended when not indicated.

*This can lead to severe bleeding events, even death* [7].

Finally, inappropriate usages might be related to **processes outside the healthcare system**. Most patients spend only a minor part of their life in front of a care provider. Most medication-related activities (taking the pills, or not) occur outside the healthcare system, at home, at work, on vacation, etc. Nevertheless, multiple health data ecosystems do not explicitly offer the possibility to store such information [8].

### 1.6. Aspirin Bought over the Counter by a Patient

While natural products are notorious for interacting with prescribed health treatments, multiple drug products can also be bought over the counter. The availability will vary significantly from one jurisdiction to another, some allowing unrestricted access to antibiotics, for example.

*This could lead to important bleeding with an emergent neurosurgical procedure which might be done differently if knowing the patient takes aspirin.*

### 1.7. Morphine 5 mg PO QID PRN

Even when the intentions of a care giver are aligned with best practices, a certain degree of abuse can occur.

Individuals can forge prescriptions or copy an original one and go around multiple pharmacies, sometimes in different jurisdictions. Taking the measure of this and acting on it requires a full view of data in the chain of drug-related activities, physicians, nurses, and pharmacists.

*This can lead to both individual and populational problems like the opioid crisis present in multiple jurisdictions.*

We will use these challenges to illustrate how a proper semantic model could contribute to the prevention of these problematic situations. The current work aims at presenting such a model: PDRO 2.0 (the Prescription of DRugs Ontology 2.0). The method section will briefly survey existing approaches to data integration and describe how ontologies can achieve this goal. The result section will present the salient classes of PDRO 2.0. This is followed by a discussion where the ontological entities will be related to the problems described in the introduction in order to illustrate how PDRO can address them.

## 2. Methods

These challenges present significant considerations to guide potential solutions. Firstly, solutions will need to be able to cope with data from varied and geographically distant jurisdictions. Patients need to travel long distances to seek very specialized outpatient care but will purchase their medication back home. Some problems are populational. Understanding the roots and tracking the results of interventions require a large perimeter. The problems also highlight the need to integrate data on a large scale both in terms of volume but also domains/systems well beyond what is traditionally identified with the pharmaceutical domain. Given these requirements, aiming at building one central system storing all the required data to address all these problems is not feasible for various reasons, among them: political, legal, ethical, or social considerations.

A solution must therefore enable multiple, heterogenous systems to contribute to the unified view of an individual, that is without requiring a copy of all data to a central database. This requires:

- Lifting all ambiguities to enable correct alignment between the various systems and the view.
- The possibility to align data in multiple domains to form a coherent model.

Ideally, given the scale required and the technological implications of creating such a virtual view, the chosen model should be verifiable for both consistency and coherence. Another desired function might be to reason on the model and aligned data in order to discover new relationships.

Integrating data from multiple systems presents various considerations, one of them being interoperability. The interoperability problem has been described as the Babel tower problem [9]. This stems from multiple sources having different technical, syntactic, and semantic ways to store, structure, exchange, and define data about connected domains. One of the first approaches to tackle this has been the use of common data models. The idea was to make sure everyone used the same system. Obviously, given the problem at hand, this would not be feasible. However, the common data model (CDM) approach can also be used differently. One can translate data from local systems through a process called ETL (for extract transform and load) and generate a new version of the data. That “copied” version is using the CDM at each site. It can therefore be assembled in subsequent steps. 

### 2.1. Common Data Models

This approach has been used successfully in research networks and groups of similar organizations, for example *Informatics for Integrating Biology & the Bedside* (I2B2) or *Observational Medical Outcomes Partnership* (OMOP) [10,11]. While interesting, these CDM would not cover the extent of data required here (including quantified self-data capturing, such as respiratory rates from a watch, or drug administration records from an automated system to improve compliance). This also implies copying data to a new model. Given the multiplicity of CDM, an organization might end up with tens of copies of its data to be able to participate in all relevant initiatives. This hinders scalability. Significant resources must be expanded to keep all these systems up to date and copied data synchronized. This approach also puts the emphasis on data structures rather than data semantics. This can lead to logical inconsistencies that are difficult to identify using only the CDM. While outside the scope of this article, a specific example of these inconsistencies, the counting of entities problem with the OMOP CDM can be found here [12].

Another approach is to focus on the messages by which data is exchanged and different options have been proposed in that regard. One such approach is the Fast Healthcare Interoperability Resources (FHIR) [13]. This follows in the footsteps of previous versions of HL7 standards like the v2 and v3 versions. Even if largely adopted in multiple jurisdictions, its scope being dedicated to the healthcare system, large amounts of data reside elsewhere. More fundamentally, here too, semantic problems arise as they try to extend it to other domains like veterinary medicine. For example, the patient resource [14] is defined as “Demographics and other administrative information about an individual or animal receiving care or other health-related services”. It has a Patient.name attribute taking the value type “HumanName”, while HumanName is defined as “A name of a human with text, parts and usage information”. Therefore, if the patient resource is used in a veterinary setting, one could deduct that the name of an animal is the name of a human. Describing entities by the way they are documented (syntax) rather than by axiom and rules relating to what they represent (semantics) creates models that can lead to inconsistencies and ultimately wrong deductions [15]. 

### 2.2. Ontologies

Ontologies, on the other hand, primarily focus on semantics. It is the representation of a set of entities and their relationships in a particular domain by means of classes and properties [9]. An ontology is expressed through language that respects a description logic which allows the representation of the domain according to a formalism that is both understandable by human experts and computable by a computer. Multiple technological implementations and syntaxes coexist in the space and are chosen for pragmatic reasons but can be translated from one to the other without loss. One of the most common ontological standards is OWL 2, a W3C standard [16]. This use of description logics brings important benefits. Complex relationships can be described formally both in terms of logic and semantics. This in turn enables reasoning both on the classes themselves (to verify the coherence of the model) and the data (instances) to discover new relationships between data pieces.

Ontologies are different than terminologies. Multiple terminologies exist worldwide in the biomedical domain, including the Unified Medical Language System (National Library of Medicine, Bethesda, MD, USA) that relate different terminologies representing the same concepts (e.g., diabetes), or the Health Canada drug product database containing codes for the available commercial drug products. Nevertheless, these resources do not describe the structure of biomedical documents nor the rules governing their subparts (mereology). Terminologies consequently do not, by themselves, enable error checking (is there an important missing part, a contradiction, etc.) nor a translation path from one system format of documents to another. They can be used in conjunction with ontologies though, so that free text is limited and translations are more consistent, for example between languages.

The use of ontologies to structure international biomedical work is now well established, with large initiatives like the Open Biological and Biomedical Ontology (OBO) Foundry [17]. The foundry has the mission of steering the generation of a family of orthogonal (compatible and not overlapping) ontologies that are both logically and scientifically well formed to describe the biomedical domain. Duplication is minimized by community submissions before being accepted in the foundry. Prime examples have already been deployed on large scale like the Gene Ontology [18]. It is also important to note that most ontologies are open source, including for commercial use. 

It follows that ontologies are not information systems or data access platforms themselves. However, data access and sharing platforms using ontologies as backbones have been successfully deployed in multiple domains [19,20]. This is also the case in the health domain. For example, the TRANSFoRm (a European commission FP7 funded project) used an ontology [21,22] to facilitate data access as part of research projects in primary care in Europe (a challenging environment with multiple small organizations having limited resources). More recently, the PARS3 platform has been developed to support learning health system activities, including physician’s reflective practice (using electronic medical records, hospital emergency visits data, etc.) or rare disease research [23].

## 3. Results

As part of the clinical knowledge model used by PARS3, we have developed several domain ontologies for domains such as drug prescriptions with the prescription of drugs ontology PDRO [24,25], or laboratory test reports with the clinical laboratory ontology LABO [26]. Following the OBO Foundry methodology, we built these ontologies upon pre-existing ones from the OBO Foundry, including high-level, foundational ontologies like the Basic Formal ontology (BFO [27]), mid-level ontologies such as the Information Artifact Ontology (IAO [28]) or the Ontology for Biomedical Investigations (OBI [29]). This allowed us to import the following classes:

- OBI: *Planned process* which encompasses processes (something that happens) that follow a plan, such as giving a drug to a patient.
- IAO: *Information content entity* (ICE) which represents pieces of information, independently of their medium.
- IAO: *Directive information entity* (DIE), a subclass of ICE that represents ICEs that aim to direct a planned process.

Based on the above classes, the PDRO 2.0 ontology proposes several classes and axioms that are relevant to medication management. The classes among them, that are paramount in addressing the previously defined problematic, are described below.

The starting point of a drug administration to a patient is generally a prescription, which is represented by the class *Drug prescription*, defined as follows:

- *Drug prescription* = def. “A health care prescription specifying the initiation, modification or cessation of a drug administration”.

The definition of *Drug prescription* mentions a drug administration. We consider a drug administration to be the process of administering a collection of drug doses, as it consists of a set of individual dose administrations. While a drug administration is usually directed by a prescription, this is not always the case. Therefore, we propose the following classes:

- *Drug dose administration* = def. “A planned process that has as participants an extended organism and a drug product and that results in a specified portion of the drug product (a single dose) being located in the extended organism”.
- *Drug administration* = def. “A process that is the mereological sum of some drug dose administrations to a single extended organism”.

In a drug prescription context, the ontology allows us to define the directive entities that specifically direct these processes and thus to propose a detailed structure (or mereology) of the different components of a prescription. The building blocks of a prescription are the *Drug prescription items* which each define the modalities of administration for a given drug. It is defined as follows:

- *Drug prescription item* = def. “A directive information entity that is a part of a drug prescription and specifies some action(s) related to one or several drugs. It is intended to direct some actions to be performed by a patient and some pharmacist(s). It may also specify a healthcare objective”.

**Figure 1:**
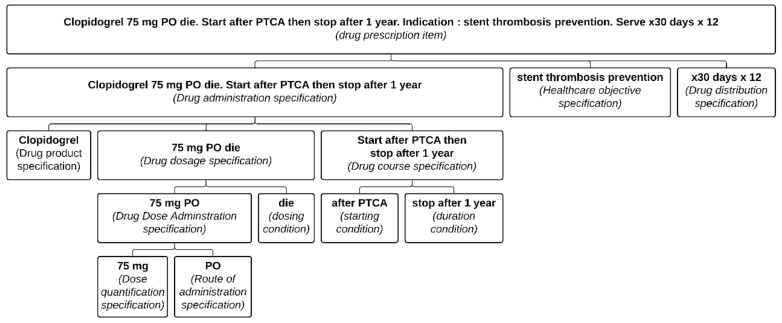
Representation of a well-defined Clopidogrel prescription item formalized in the PDRO ontology. Each part of the prescription item is associated with its relevant class. (PTCA: percutaneous transluminal coronary angioplasty; PO: per OS).

A *Drug prescription item* also includes different specifications as defined below and organized as illustrated in Figure 1.

- *Drug administration specification* = def. “A directive information entity that specifies a drug administration. It specifies at least the drug product. It often also includes a drug dosage specification and some drug course specifications”.
- *Drug course specification* = def. “A directive information entity that specifies the conditions governing the duration, initiation and termination of a drug administration”.
- *Drug dosage specification* = def. “A directive information entity that directs the dosage in a drug administration through a drug dose administration specification and often conditions required to give a dose such as frequency or periodicity”.
- *Drug dose administration specification* = def. “A directive information entity that directs the administration of a dose”.
- *Drug dispensing specification* = def. “A directive information entity that specifies the dispensing of a drug product”.

Another aspect that must be taken into account concerns the various processes that occur downstream of the prescription. They include the processing of the prescription by the pharmacist in order to dispense a given amount of a drug product in accordance with the prescription.

- *Drug prescription dispensing request processing* = def. “A planned process having as specified input some drug prescription, and that aims at generating some drug-dispensing orders based on algorithms, legal requirements, and professional judgment of the pharmacist”.

**Figure 2:**
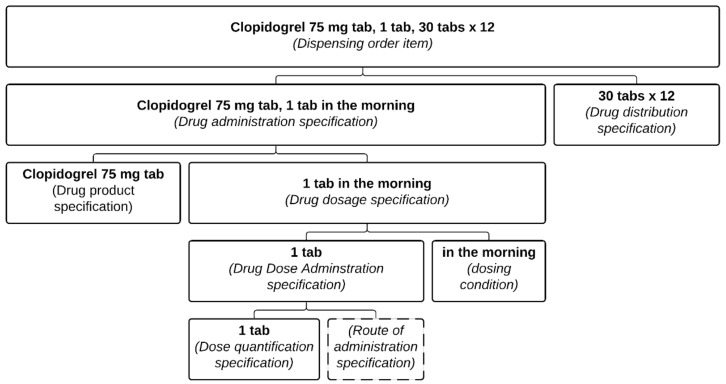
Representation of pharmacist dispensing order item. This is the result of the processing of the prescription from Figure 1. Each part of the pharmacist dispensing order item is associated with its relevant class.

**Figure 3:**
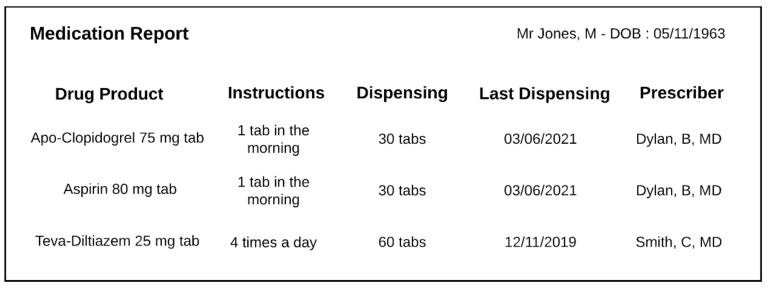
Example of a typical pharmacist dispensing report. Each line contains elements from a dispensing record item.

The structure of a drug-dispensing order (Figure 2) is similar to that of a drug prescription, insofar as a *Drug dispensing order* item includes a *Drug dispensing specification* which in turn directs a *Drug dispensing*. The particularity is that the pharmacist will also record what has been dispensed in a *Drug dispensing record item* (Figure 3). These classes are defined as follows:

- *Drug dispensing order item* = def. “A directive information entity that is a part of a dispensing order and that specifies some action(s) related to some drug products. It is typically intended to direct some actions to be performed by a patient and pharmacist(s)”.
- *Drug dispensing* = def. “A planned process in which a specified quantity of a particular drug product is made available with the goal of the drug product being administered to an organism”.
- *Drug dispensing record item* = def. “A data item that is part of a drug-dispensing record and describes how much of some drug products have been dispensed to a patient”.

This formalization allows us to distinguish for a given prescription item what has been prescribed (the *Drug administration specification*) from what has been distributed (the *Drug dispensing record item*).

## 4. Discussion

Major challenges can be identified with regards to medication usage, both at the individual and population level. The entities from PDRO described in the previous section can contribute to addressing the problems outlined in the introduction.

### 4.1. Ambiguous or Incomplete Drug Administration Specifications (DAS)

Firstly, the drug administration specifications (DAS) are not always sufficient to safely and optimally drive drug administrations. One cause is that not all necessary pieces of information to correctly assess the safety or pertinence of medication administrations can be included in a DAS [30]. 

In the ibuprofen example, two important elements are not included in the DAS. 

The first one includes information to adjust the administration if needed. This could include laboratory evaluation results (like the renal function), height or weight measurements. Here also, existing OBO foundry can be leveraged to express these data, such as the clinical LABoratory Ontology (LABO) or the Questionnaire ontology (QUESTO) [31]. 

The second important element missing from the DAS in the ibuprofen example is the reason for the drug administration (e.g., is the treatment administered for back pain or pericarditis?). Multiple formalisms such as OMOP do not include it. PDRO enables the structuration and capture of such information through its inclusion of a *health care objective specification* within the *Drug prescription item*. Since this is likely present in the electronic medical record at the time of the prescription, supporting the provider by enabling a choice of reasons should be feasible. This is especially important since drug administrations can be used for reasons other than to treat a disease; for example, they might be a part of laboratory evaluation (e.g., an ACTH suppression test where a corticosteroid is administered prior to the blood test). This can be done using classes from other OBO foundry ontologies, such as *OMGS: Diagnosis* in the Ontology of General Medical Sciences (OGMS [32]), or classes from LABO in the ACTH example above. 

**Figure 4:**
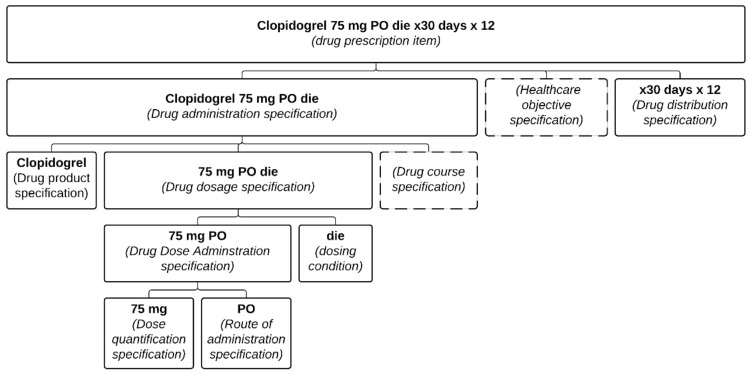
Representation of the prescription item presented in the Clopidogrel example formalized in the PDRO ontology. Each part of the prescription item is associated with its relevant class.

This was also a problem in the Clopidogrel example (see Figure 4). Had “stent thrombosis prevention” been included as a reason, the risk of error would have been greatly diminished. Yet, even with the presence of the health care objective, this still relies on some assumptions (namely that for such a situation, 12 months should be the duration). Multiple exceptions exist, and the ideal is to indicate both the reason for the treatment (as part of the DAS) and the condition describing when the medication should be stopped (see Figure 1).

Another important factor is that PDRO enables prescribers to express conditions in different terms. For example, a stopping condition could be “do not continue after you have taken 720 pills” (in the case of a medication taken twice a day). This could be wrongly assumed to be equivalent to “do not continue after a year from the prescription date”. Most patients will not comply perfectly (that is, they will not perform drug administrations exactly as specified by the prescriptions and pharmacist instructions) [33]. This will lead to intervals between renewals to be longer than in case of perfect compliance. Assuming a 50% compliance rate, the 720 pills mentioned above could be self-administered over two years instead of only one (a prescription is valid two years by default in the province of Quebec, Canada). A stopping condition of one year from the prescription or after 720 pills could therefore yield quite different drug administration processes.

Ambiguous DAS can also lead to other significant problems as with the Cardizem problem where what is on the prescription is not precise enough to correctly time the drug dose administrations. This lack of precision hinders the use of automated systems helping patients to take their medication safely and as directed. Here again, the absence of sufficient information could lead to inappropriate suggestions by automated systems or inability to use them if, for safety, they will not accept to work with ambiguous prescriptions.

The fact that ontologies have been designed to be both machine and human readable can alleviate a significant part of this problem. While it obviously cannot invent information that is not there, it can support the care providers. Firstly, PDRO can structure this information to ensure clarity and consistency. In the dosing specification, targeted time intervals with accepted variations can be structured more precisely (e.g., “12 h +/− 2 h”) Secondly, it is not expected that care providers would need to enter all of this manually. Already today, dosage specifications can be suggested to the prescriber by electronic prescribing tools, based on the chosen active ingredient. With dosage specifications structured using PDRO, a more complete, precisely structured specification could be suggested without necessitating more work from the prescriber. For example, even if a physician is used to write “Cardizem 60 mg PO QID”, the system could ask the user to enter the medication (Cardizem), the dose from a list of possibilities (60 mg) and the number of administrations per day (4) and the system could automatically generate: Cardizem 60 mg PO q6 h +/− 1 h. As a result, the physician needs to input only three pieces of information to generate a non-ambiguous prescription item which will clearly indicate to the patient that one dose will need to be taken at night. This would better guide the patient and enable the use of compliance improving tools (e.g., smartphone app reminders or smart pill bottles). 

Finally, this ambiguity can also have economic consequences at both individual (increased cost out of pocket, decreased compliance, etc.) and populational levels (insurance plans, government expenses, etc.). The exemplar prescription mentioning Gluconorm illustrates this situation. The tolerated ambiguity regarding the way to describe the drug in a DAS (through active ingredients or commercial brand names) has led authorities to enact rules authorizing or sometimes mandating substitutions for less costly drugs even when a commercial brand name is used. This ambiguity can be difficult to untangle as some commercial names use the active ingredient name. Consider for example “Metoprolol”: it so happens that it is an actual commercial drug product name from Sanis Health Inc, but also an active ingredient. Therefore, “Metoprolol 50 mg PO twice a day” is very ambiguous. PDRO can lift this ambiguity by clearly marking the semantic of the drug-related text in the DAS as a reference to an active ingredient or a reference to a specific commercial preparation.

### 4.2. False Assumptions about a Drug Administration Specification When Using Dispensing Documentation

In the context of ambiguous or missing information on prescriptions (or not having access to the prescription at all), it could be tempting to approximate the missing information using other documents like the distribution documentation at the pharmacy [34]. Given the importance of this information for financial (business profitability, insurance reimbursements), operational reasons (inventory management), and public health, this information has been historically digitized for much longer than other sources of information.

Absent, or secondary in the distribution documentation, is the clinical intent. Both Amlodipine and Clopidogrel illustrate the perils of relying solely on distribution records. Even if the initial prescription contained the reason for the prescription, multiple dispensing records would not include it. The notion that the administration had a definite end period is irrelevant from the pharmacy’s point of view, as any subsequent refills would have to be prescribed anew anyway. Take, for example, the prescriptions on Figure 1 and Figure 4, which could both lead to the same dispensing order (Figure 2). Therefore, for another physician than the original prescriber consulting the pharmacy records, the original intent of the prescription is no longer apparent and may lead to an inappropriate renewal during broad re-prescription of a patient’s medication list. 

A tempting solution to the lack of prescription information would be to enforce that the prescriptions presented at the pharmacy for distribution must be recorded in full alongside pharmacy dispensing records. While worthwhile, this approach would still be insufficient. Prescriptions hold clinically relevant information whether they reach a pharmacy or not and should be documented independently. Patient compliance cannot be adequately appreciated solely from distribution records. For a physician treating a patient, the knowledge that a medication was prescribed by another physician and that this prescription was not taken to a pharmacy is valuable. Furthermore, prescriptions aiming at ending medication (“stop Ativan”) may never reach a pharmacy because most patients may expect that non-action will lead to its cessation at the pharmacy. For example, distribution records are ambiguous if Ativan is in a status of “not renewed” and the clinical intent of stopping it (for whatever reason) is absent. This could be because the patient has no more insomnia or because she was told to stop it by her physician and simply never went back to the pharmacy. The use of independent documentation of prescriptions and distributions, as described in PDRO 2.0, can completely describe these issues.

Finally, empirically, previous experience in automated surveillance systems for potential inappropriate prescriptions [35] has shown that, taken alone, drug distribution information is limited in its discriminatory power to arbitrate if a treatment is appropriate or not. Clinical care (helped by algorithms or not) could benefit greatly from the added information provided by other health care data such as medical history, laboratory tests results, habitus, or quantified self. Being able to follow weight and diuretic use in heart failure or hypotension episodes would certainly enable a much more personalized approach to medication monitoring. The orthogonal set of ontologies in the OBO Foundry can contribute to and facilitate the advent of this vision.

### 4.3. Outside the Healthcare System

Relying solely on information detailing prescriptions or even distributions can lead to an incomplete picture. Many products are available without prescriptions, some can be ordered from outside the jurisdiction and “natural” products need to also be taken into account as they can interact with prescribed drug products (e.g., St. John’s wort and clopidogrel) [36].

Many health processes occur outside the healthcare system but can have important consequences on care delivery in the system as illustrated by the Aspirin example. This is why PDRO 2.0 is carefully attached to root classes bringing together the processes both in and outside the healthcare system, particularly regarding drug administration.

Health activities outside the healthcare system might also include undesirable events. The definition of *Health activity* has been created explicitly to take into account activities which would not improve someone’s health status, for example COVID-19 exposure as part of a trial drug administration outside a prescribed setting. 

Drug misuse is a major public health concern. In response to the opioid crisis, electronic prescription drug monitoring programs (PDMPs) were set up in multiple jurisdictions (ref…). The agglomerated data is a means to curb doctor and pharmacy shopping [37]. They generally rely on drug-dispensing data and algorithms to identify patients at risk. Unfortunately, the limited information available can lead to false positives, especially in the case of cancer patients [38]. 

Another important caveat to PDMPs is the duplication of information. As a matter of fact, their functioning relies on existing databases (from pharmacies) sending relevant dispensing data to a central database. Care must be taken as corrections or changes to the initial data must also be applied to the data stored centrally to maintain integrity. Additionally, PDMPs require advanced knowledge of relevant data elements to be transmitted. Through a common ontology, querying existing databases in their native data representations enables dynamic interrogations of available medical data without the need for duplication or prior knowledge.

## 5. Conclusions

This work demonstrates that optimizing drug prescribing and improving medication management requires a systemic view with the virtual integration of data from multiple heterogenous systems across wide geographical areas and from multiple domains above and beyond pharmacies. Ontologies can support this task and technical implementations exist to leverage ontological models and deliver a unified view of the data for further analysis or care delivery. 

Regarding the ontological model itself, PDRO 2.0 supports the disambiguation of prescriptions, both at the drug administration specification level and for complementary information. It is built according to the OBO Foundry principles in order to facilitate the integration of information from other domains like the environment (ENVO [39]) or genomics (e.g., GO, the Gene Ontology [18]). Proper relations enable the relation of prescriptions to their distribution records when appropriate and processes outside the healthcare system can also be taken into account seamlessly.

Simply using PDRO will not by itself make information that was not captured appear magically. It can nevertheless enable the annotation of existing data when the implicit intent can be made explicit and it can guide further data creation to ensure maximal explicit information at the time of its generation. Maybe as important, it can clarify the relations and definitions of different data artifacts in order to minimize the risk of drawing incorrect conclusions, both at the individual and populational levels.

While annotating existing data is a first step, further work is underway to explore the possibility of using PDRO to guide dynamically the creation of well formed, non-ambiguous, sufficiently specified prescriptions, distribution records and administration records.

## Data Availability

No clinical data used. Data sharing is not applicable to this article.
